# An Approach to the Design and Development of an Accredited Continuing Professional Development e-Learning Module on Virtual Care

**DOI:** 10.2196/52906

**Published:** 2024-08-08

**Authors:** Vernon Curran, Robert Glynn, Cindy Whitton, Ann Hollett

**Affiliations:** 1 Faculty of Medicine, Memorial University of Newfoundland, St John’s, NL, Canada

**Keywords:** virtual care, continuing professional development, needs assessment, remote care, medical education, continuing medical education, CME, CPD, PD, professional development, integration, implementation, training, eHealth, e-health, telehealth, telemedicine, ICT, information and communication technology, provider, providers, healthcare professional, healthcare professionals, accreditation, instructional, teaching, module, modules, e-learning, eLearning, online learning, distance learning

## Abstract

Virtual care appointments expanded rapidly during COVID-19 out of necessity and to enable access and continuity of care for many patients. While previous work has explored health care providers’ experiences with telehealth usage on small-scale projects, the broad-level adoption of virtual care during the pandemic has expounded opportunities for a better understanding of how to enhance the integration of telehealth as a regular mode of health care services delivery. Training and education for health care providers on the effective use of virtual care technologies are factors that can help facilitate improved adoption and use. We describe our approach to designing and developing an accredited continuing professional development (CPD) program using e-learning technologies to foster better knowledge and comfort among health care providers with the use of virtual care technologies. First, we discuss our approach to undertaking a systematic needs assessment study using a survey questionnaire of providers, key informant interviews, and a patient focus group. Next, we describe our steps in consulting with key stakeholder groups in the health system and arranging committees to inform the design of the program and address accreditation requirements. The instructional design features and aspects of the e-learning module are then described in depth, and our plan for evaluating the program is shared as well. As a CPD modality, e-learning offers the opportunity to enhance access to timely continuing professional education for health care providers who may be geographically dispersed across rural and remote communities.

## Introduction

Most provincial health care systems across Canada responded to the COVID-19 pandemic with a rapid adoption of digital tools and technologies, including virtual care appointments. The Canadian Institute for Health Information (CIHI) reported that between March to September 2020, the percentage of patients availing virtual care services increased from 6% to 56% [1]. Virtual care refers to the delivery of health care services digitally or at a distance using information and communications technology [2-4]. During COVID-19, a variety of virtual care types were used, with synchronous and asynchronous appointments being the most common [3,4]. Synchronous virtual care refers to communication between the health care provider and patient that occurs in real time and can include the use of telephone or videoconferencing. Asynchronous communication does not occur live and may include the use of e-mail messaging, messages left for patients in a portal site, and e-consultations [3-5]. Considering the goal of reducing COVID-19 exposure during the recent global pandemic, virtual care proved to be most effective in that it minimized exposure and risk to health care providers by reducing the movement of people [2-7]. In addition, virtual care helped patients stay home who may have otherwise traveled to a health care site and incurred the risk of unnecessary exposure [3,4,6,7]. Virtual care was also used for control and triage during COVID-19, remote monitoring of patients, treatment and management, and provision of online health services [3,4].

In Canada, considerable work during and after the COVID-19 pandemic was undertaken to develop guidelines to inform physicians, health care providers, and patients on how they could best use virtual care. The Canadian Medical Association (CMA) and Royal College of Physicians and Surgeons of Canada (RCPSC) developed resources for both physicians and health care providers, as well as patients. The “Virtual Care Playbook” provided virtual care guidance for providers and connected patients to the “Virtual Care Guidelines for Patients” [8,9]. Canada Health Infoway has also undertaken significant work regarding virtual care support for physicians and health care providers. In particular, Infoway’s “Clinician Change Management” project provided support in the form of virtual care tools and training [10]. The Canadian Medical Protective Association (CMPA) has also supported providers by disseminating virtual care informational resources for physicians and health care providers through their website [11].

With the rapid introduction of virtual care across many jurisdictions during the COVID-19 pandemic, both health care providers and patients alike were not always adequately trained on how to use virtual care appropriately. Previous research has suggested that a lack of training around virtual care tools and software was a challenge for providers. A lack of understanding and training may have contributed to lower confidence levels among providers and a reluctance to use virtual care, thereby negatively influencing virtual care adoption [3,4,12-15]. Adjusting clinical approaches to caring for patients remotely can also be challenging, including how to virtually examine patients by videoconferencing or telephony systems [3,4,16,17]. The use of new digital health systems like virtual care also requires knowledge and competence in how to incorporate the technology within a provider’s practice workflow. This includes understanding how to use the technology effectively, as well as the privacy and security issues surrounding the use of the technology. Providers also need to be able to adapt their techniques and clinical acumen to build rapport with their patients while using virtual care technologies. Given this, consideration of the potential continuing professional development (CPD) needs of health care providers is critical to ensuring that proper support systems and training are available to enable and empower providers to adopt and use virtual care most effectively and efficiently.

e-Learning has been defined as any educational intervention mediated electronically via the internet [18] and has become a popular modality for providing CPD in the health professions, with offerings across a diverse array of topics and subject areas [18,19]. The advantages and benefits of e-learning have been described as including lower costs, widespread distribution, increased accessibility to information, frequent content updates, and personalized instruction in terms of content and pace of learning [18]. Several systematic reviews of e-learning effectiveness in health professions’ education, including CPD, have been published. Key findings of these reviews suggest that e-learning is associated with large positive effects when compared with no interventions [20]; e-learning can be as effective as traditional methods of teaching and instruction [20-22]; e-learning and traditional educational interventions take similar time to participate in or complete [23]; and interactivity, practice exercises, repetition, and feedback are important design features of effective e-learning approaches and appear to be associated with improved learning outcomes [23].

CPD encompasses the multiple educational and developmental activities pursued by health care providers to maintain and enhance their knowledge, skills, performance, and relationships in the provision of health care [4,24]. For many regulated health care providers around the world, CPD participation is often mandated and required throughout the extensive postlicensure phase of the provider’s career. It is viewed as a key means for providers to stay current and up to date with evidence-based practices in their professional field. The evidence for CPD participation suggests that health care providers who participate in formal CPD activities are more likely to provide better care than their peers who do not participate [4,25]. CPD, which is designed to be interactive, practice-based, and longitudinal in nature, is also believed to yield better outcomes [4,25]. A needs assessment–driven approach to the development of CPD is more likely to lead to a change in practice, largely as a result of the learning being directly linked to personal and practice needs [4,26].

Edirippulige and Armfield [4,27] reviewed a number of studies describing the delivery and evaluation of education and training in telehealth. They identified 9 peer-reviewed studies describing education and training in telehealth that included several CPD-level courses on telehealth. Online learning was the most common delivery format described across the studies, with course duration ranging from 1 week to 6 months [4]. More recently, several studies conducted during the COVID-19 pandemic have reported on CPD in virtual care also using online delivery formats [28-31]. Both synchronous and asynchronous modalities were used in providing CPD on virtual care or telehealth; however, the most common delivery format was the use of web conferencing (eg, Zoom and Skype). Topics covered across these programs included introduction to virtual care, advantages and disadvantages of virtual care, types of virtual care, ensuring privacy during appointments, and legal and technological requirements for virtual care. One interesting method described by Hayden et al [30] was the use of web conferencing to facilitate simulated telehealth appointments with standardized patients. Participants found the use of standardized patients to simulate a virtual care appointment enhanced their confidence in focused telehealth skills. The use of online learning formats was perceived favorably by participants across the studies and was found to be particularly useful in accommodating the busy schedules of providers [28].

The purpose of this paper is to describe our efforts to design and develop an accredited e-learning CPD module on virtual care for physicians and health care providers. First, we discuss our approach to systematically exploring the needs of health care providers in learning to use virtual care effectively and efficiently in their practices. We describe results from a survey questionnaire we administered to a sample of health care providers in Newfoundland and Labrador, Canada, findings from key informant interviews with several experts in virtual care, and key themes emerging from a focus group with patient representatives. We then describe our approach to designing and developing this e-learning module, including key interactivity and design features to foster effective learning. Finally, we describe our approach and plan to evaluate the effectiveness and impact of this e-learning module on health care providers’ adoption and use of virtual care. The work described in the paper was undertaken by our team with the Office of Professional & Educational Development (OPED), Faculty of Medicine, Memorial University of Newfoundland. The Faculty of Medicine at Memorial University has long been a pioneer in research and development in the fields of telemedicine, tele-education, and digital learning for physicians and rural health care providers. Our Professional Development office was one of the first CPD units in North America to introduce accredited e-learning programming for physicians through our MDcme [32] learning management platform [33].

## Methods

### Needs Assessment Study

We undertook a needs assessment study initially as a first phase of our project to design and develop an e-learning CPD module on virtual care. The needs assessment encompassed a web-based survey, key informant interviews with experts in virtual care, and a focus group with patient representatives [3,4,34]. The goal of the web-based survey was to explore the experiences, perceptions, and satisfaction of health care providers with the adoption and use of virtual care during COVID-19. We developed and distributed this survey to physicians, nurses, and allied health professionals across the province of Newfoundland and Labrador, Canada, to explore their CPD needs and preferences as well.

In total, 51% of respondents (n=432) in our survey indicated they were currently offering virtual care and a majority (68.9%) reported it had improved their work experience [3]. The telephone was the most used method and respondents reported the most comfort and satisfaction with telephone appointments [3]. The most challenging aspects of telephone appointments were the inability to conduct physical exams to the degree required, the inability to assess physical health status, and the patient’s or client’s cell phone service being unreliable [3]. Respondents rated the importance of a variety of CPD topics on effective use of virtual care, and the highest rated topics included compliance with regulatory standards or rules for virtual care, understanding boundaries (eg, personal telephone numbers used to call patients or clients), and developing and maintaining competency and professionalism while engaging in virtual care [3]. Other important topics for virtual care CPD included CPD on how to use the technology, the best or easiest platforms for providing virtual care and how to use them effectively, and assessment skills and aids for doing assessments virtually. Ethical issues and legalities of virtual care were also identified by respondents as valuable as well [3].

The second component of our needs assessment was a qualitative study to explore experts’ ascribed opinions on health care providers’ CPD needs in virtual care [4]. We conducted semistructured interviews with a purposive sample of key informants representing Canadian provincial and national organizations with expertise in virtual care delivery. According to the key informant respondents, lack of training specific to virtual care tools and software was a challenge for health care providers, particularly videoconferencing appointments. All key informants identified technology as a main barrier or challenge, not only for health care providers but also for administrative staff. The main areas of knowledge, skills, and abilities deemed most important for health care providers in adopting and using virtual care identified by the key informants included effective use of technology, knowledge of how to integrate technology and virtual care in the practice workflow, privacy and security aspects of the technology, and adaptation of examination skills to virtual care and how to build effective rapport with patients [4].

A focus group study was also conducted with a purposive sample of patient representatives to explore patients’ experiences and perspectives on the adoption and use of virtual care during COVID-19, and identify the education and informational needs of patients [34]. The findings from the patient focus group were useful in informing the types of topics to include in CPD on virtual care. Patient respondents felt that virtual care was beneficial and enabled greater convenience, flexibility, and access to health care services. Key barriers and challenges in adopting and using virtual care appeared to primarily arise from patients’ lack of knowledge, understanding, and familiarity with it. Cost, technological access, connectivity, and low digital literacy were challenges for some patients, particularly in rural communities and among older patients. Patient education and support were critical and needed to be inclusive, easy to understand, and include information regarding privacy, security, consent, and the technology itself.

### Approach to Mainpro+ and MainCert Accreditations for CPD Credit

The OPED, Faculty of Medicine at Memorial University is an accredited provider of CPD that targets the needs and competency development of health care providers within Newfoundland and Labrador and beyond. OPED is an accredited provider of university CPD by the Committee on Accreditation of Continuing Medical Education (CACME) and the Association of Faculties of Medicine of Canada (AFMC). As an accredited CPD provider, OPED is permitted to accredit CPD activities that meet the administrative, educational, and ethical standards of the College of Family Physicians of Canada (CFPC) Mainpro+ Certification program [35] and the RCPSC Maintenance of Certification program [36]. Key requirements for accrediting CPD activities include a needs assessment and the formation of a scientific planning committee (SPC) to oversee and advise on the development of the accredited CPD activity. An SPC is a group of target audience representatives responsible for identifying the educational needs of the intended target audience; developing educational objectives; selecting educational methods; selecting speakers, moderators, facilitators, and/or authors; developing and delivering content; and evaluating the outcomes of an accredited CPD activity. Requirements for accredited e-learning activities also include a means for participants to interact with the material, with each other, and with faculty members or a facilitator and the ability for participants to track their progress, provide evaluation feedback, register, and receive a record of registration. Such programs must also be offered within a definitive period of time communicated before the start of the program.

### e-Learning Module Design

We ensured our e-learning module met the requirements of Newfoundland and Labrador’s primary health care providers by establishing 2 guiding committees during its design and development. The first, an advisory committee, ensured alignment with policy and practices within the provincial health care system. This committee included representation from the Newfoundland and Labrador Centre for Health Information, the provincial government’s department of health, Memorial University’s Faculty of Medicine, the College of Physicians and Surgeons of Newfoundland and Labrador, the College of Registered Nurses of Newfoundland and Labrador, and the Newfoundland and Labrador Medical Association. The second committee structure, an SPC, oversaw the design and development of the module and was responsible for ensuring that the learning experience reflected the needs of the primary health care providers [35]. This committee included a family physician, registered nurse, nurse practitioner, specialist physician, and emergency medicine physician.

We provided the advisory committee with the information collected through our needs assessment process and asked the members to offer feedback in terms of system-level needs. We then engaged with the SPC to review the needs assessment findings and advisory committee feedback and to develop a set of learning objectives we would use to guide the development of the module. Next, we engaged with several subject matter experts in virtual care to draft instructional materials and activities that would enable us to meet our stated objectives. This instructional material was used to develop a prototype of the e-learning module that we shared with the SPC and advisory committee for review and feedback. We compiled the feedback received and adjusted the prototype accordingly. We then proceeded to launch the prototype on the MDcme learning platform, a proprietary learning management system developed by OPED to house our accredited CPD activities. The MDcme environment provides user registration, asynchronous communications, technical support, and transcript or certificate issuance. The module is developed as a series of web pages using PHP (Hypertext Preprocessor) scripting and leverages responsive design to adapt its presentation based on the device used to access. The module will undergo an annual review process during which the assessment and evaluation data are reviewed, and any requisite modifications will be made, including updates and modifications to content and approach.

Van Hecke et al [37] developed the Criteria for Reporting on Development and Evaluation of Professional Training interventions in Healthcare (CRe-DEPTH) as a way to systematically report on the development and evaluation of training interventions for health care professionals. These criteria consist of 12 items representing 4 categories, which are the development of the training, characteristics of the training, characteristics of the providers, and assessment of the training outcomes. The following description of the e-learning module on virtual care outlines aspects of the development and evaluation of this educational program according to these criteria.

In developing the e-learning module, we followed the phases of the ADDIE model of instructional design. The ADDIE model is a systematic instructional design framework widely used in the creation and development of educational and training programs. The acronym “ADDIE“ stands for 5 sequential key stages in the instructional design process, which are Analysis, Design, Development, Implementation, and Evaluation [38]. While sometimes criticized as being too linear in its approach, we have found that this framework delivers a consistent approach to educational development and aligns well with the requirements of the Mainpro+ and Maintenance of Certification accreditation programs. Gagne’s “Nine Events of Instruction“ model was also followed as an overarching approach in the development of content for the module [39].

We adopted an asynchronous e-learning design for this module. The asynchronous model assumes that learners taking the program will access the content at different times and from different locations. This approach allows primary care providers across the province to access the instructional material at their convenience, thereby providing the flexibility needed to balance professional learning with varied work hours, family, and other personal or professional commitments [40-42]. Results from our needs assessment survey of potential participants indicated that a large proportion of survey respondents preferred “E-Modules (self-paced/online learning)” as the delivery format [3].

Learners access the module by creating an account on the MDcme platform. The e-learning module provides a 90-minute introduction to the delivery of virtual care in a primary care setting and addresses the learning objectives, which are: (1) describe the benefits and key considerations of conducting virtual care appointments; (2) identify the technological requirements and setup required to conduct optimal virtual care; (3) recognize how to integrate virtual care delivery into your existing practice workflows; (4) discuss the clinical implications for delivering optimal virtual care encounters; (5) explain how to prepare patients for virtual care sessions; and (6) summarize the key regulatory and legal considerations in providing virtual care in Newfoundland and Labrador.

Our experience has been that a 60- to 90-minute duration for online CPD modules is an appropriate length to increase completion rates and reduce participant attrition.

**Figure 1.**
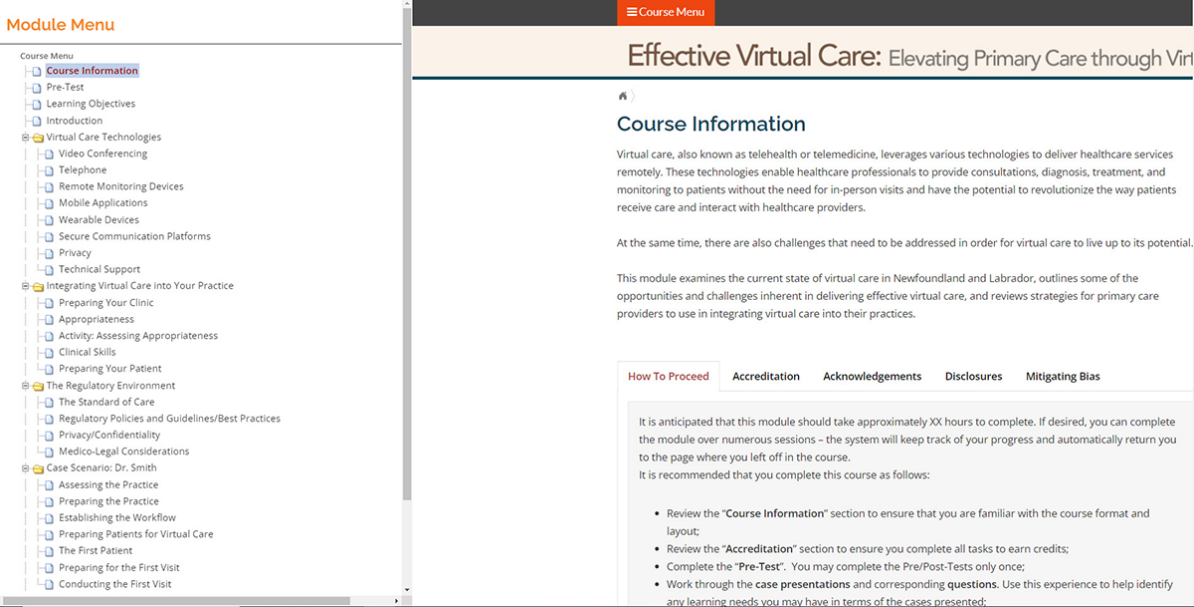
Menu interface.

The module is organized into three primary sections: (1) virtual care technologies, (2) the incorporation of virtual care into one’s practice, and (3) the regulatory landscape. While the content is structured in a sequential fashion for learners to progress through, a comprehensive course menu tree is available, enabling learners to access any section of the module whenever they wish (Figure 1).

**Figure 2.**
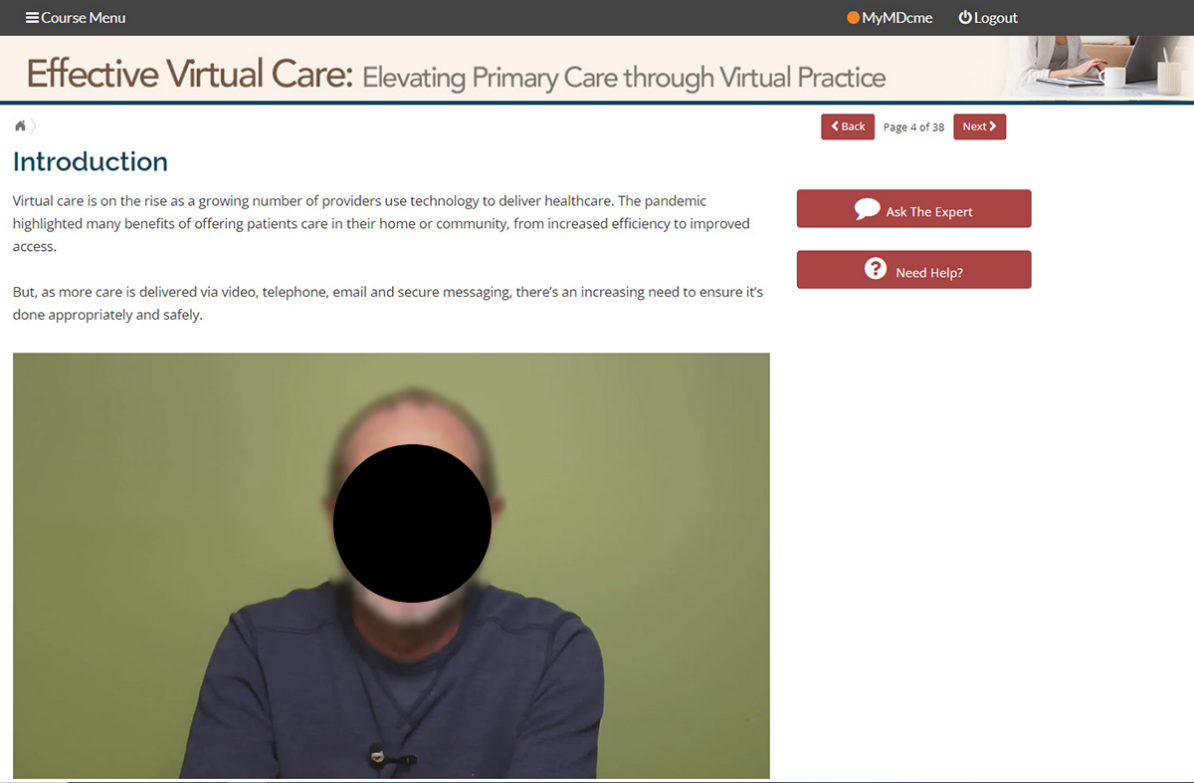
Example of video tutorial.

The module design uses several strategies to enhance learner engagement and support multimodal learning [43-45]. First, a variety of media are used in the presentation of module content, including text, images or graphics, and short video clips (Figure 2). Second, user interface design elements such as clickable tabs and dropdowns or flyouts are used where appropriate to encourage the learner to physically interact with the module. Finally, several interactive instructional design components are included, such as pre- and posttest assessments and interactive case scenarios. Several accessibility standards are also included in module design, including the use of descriptive alt tags for all graphical elements and the inclusion of closed captions for all audio or video elements for people with hearing impediments.

**Figure 3.**
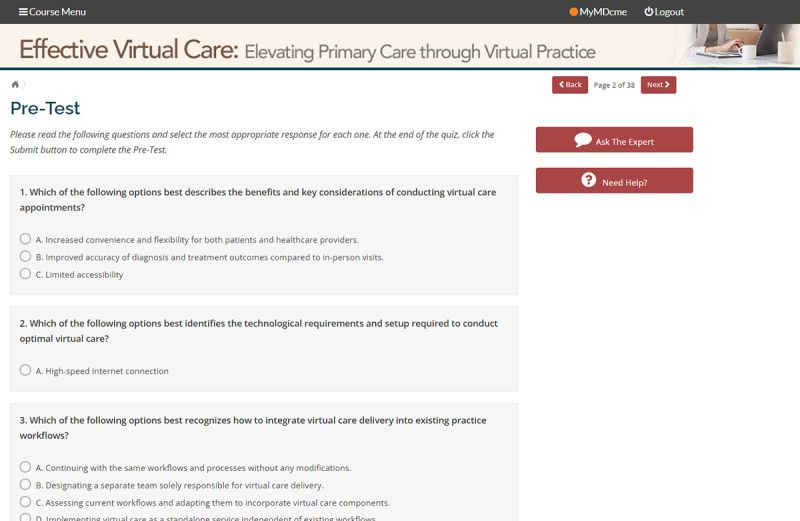
Learning assessment.

The pre- and posttest assessments are interactive quizzes that present a number of multiple choice questions designed to evaluate learner knowledge of the subject matter and enable self-assessment and reflection, as well as several Likert scale measures of learner confidence in performing the learning objectives (Figure 3). The assessment is presented once at the beginning of the module and then again at the end of the learning experience. The learner receives immediate feedback after submitting each assessment; correct or incorrect data are presented as feedback to the pretest, and correct or incorrect data along with a brief rationale for the correct response are presented as feedback to the posttest.

**Figure 4.**
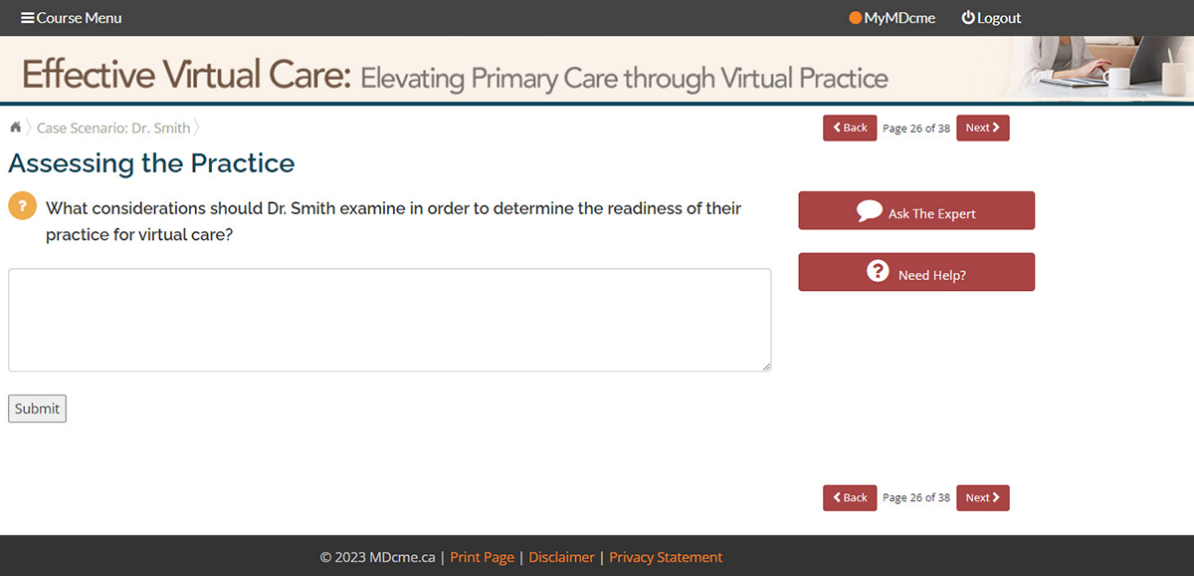
Interactive case scenario.

**Figure 5.**
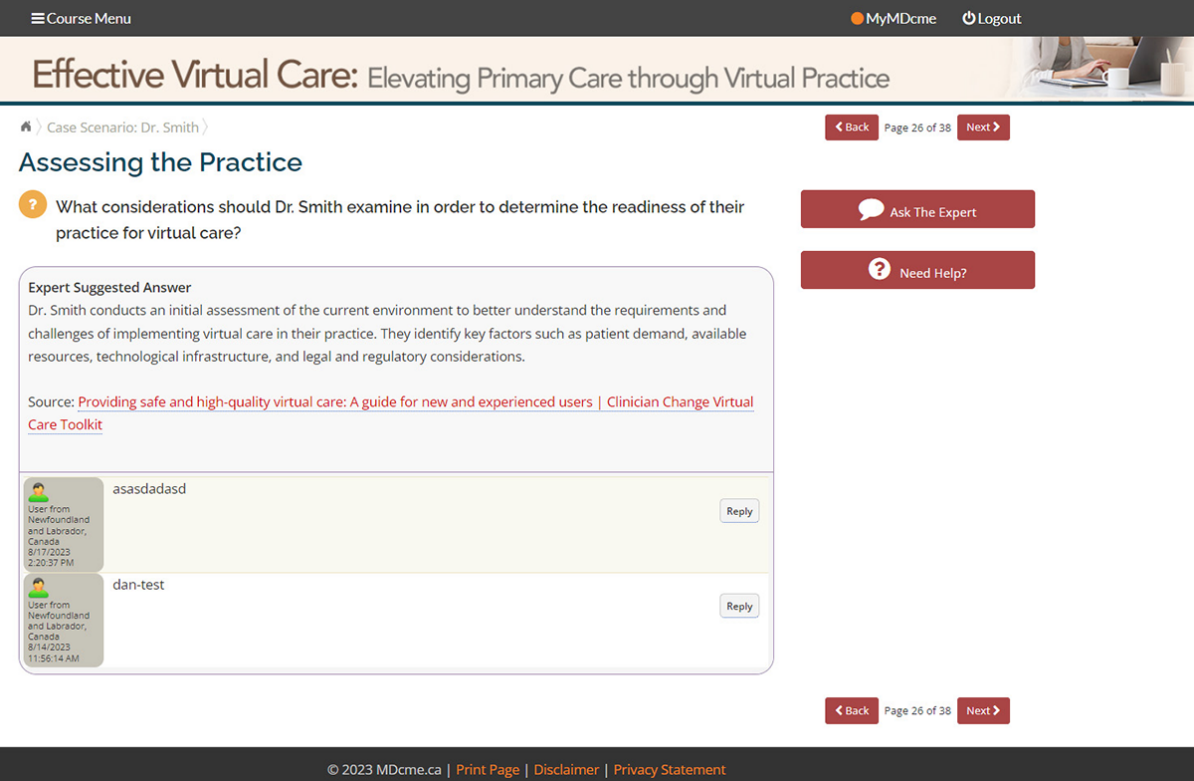
Interactive case scenario (continued).

An interactive case scenario is presented as a final learning activity in the module (Figure 4 and Figure 5). The case scenario models the application of module content to the primary care practice context. The learner is first presented with an overall scenario and then asked a series of “What would you do?” questions designed to prompt reflection. The learner enters their response and is presented with immediate feedback including the response of peer learners in the system as well as a model answer summarizing how the concepts covered in the module could be applied to the given situation.

Given the asynchronous approach used in the module design, learners are able to view peer responses to the interactive case scenario but are not able to engage in a dialog with other learners taking the course. Learners can interact with subject matter experts if they have questions related to the content presented in the module. In that case, a learner can enter a question or comment through the “Ask the Expert” feature in the module and will receive a response via email within 48 hours.

### Evaluation Approach

An evaluation approach has been designed around Curran and Fleet’s [46] use of Kirkpatrick’s levels of evaluation. The levels comprising the evaluation approach of the e-learning module include pre- and postknowledge and confidence assessments, satisfaction surveys, and a postmodule outcomes survey.

#### Pre- and Postknowledge and Confidence Assessments

A pre- and posttest assessment is embedded directly into the e-learning module. The assessment includes measures of both learner knowledge of the content covered and learner confidence in the ability to achieve the stated learning objectives. The knowledge items were developed by content experts and consist of a bank of one-best-answer multiple-choice questions. The confidence items consist of several statements related to the learning objectives for the online module.

#### Satisfaction Surveys

An online satisfaction survey is provided to the learner at the end of the module. A combination of close- and open-ended questions related to the module content and overall impressions are used to gauge satisfaction and to allow for continuous improvement of subsequent deliveries of the program. The survey enables participants to provide feedback on the module related to relevancy, appropriateness, practicality of the content, and whether they would recommend the module to others.

#### Postmodule Outcomes Survey

An online survey will be distributed to participants 6-8 months after the completion of the module. The purpose of this survey will be to further explore the impact of participation in the module on participants’ adoption and use of virtual care in their practice.

## Discussion

Through our systematic needs assessment study, we were able to specify several areas of knowledge, skills, and/or abilities that would be most helpful for physicians and health care providers as they sought to adopt and use virtual care in their practices and patient care. Respondents highlighted 3 main areas. First, the use of technology necessitates knowledge of how to integrate technology and virtual care into the practice workflow. This includes knowing how to use technology and knowledge relating to the privacy and security of the systems being used. There is an increased emphasis for providers to ensure they are meeting the standard of care, adequately obtaining consent, and embracing values of equity and fairness. Next, respondents identified the importance of being able to adapt clinical skills to virtual care and building rapport through good communication with patients. Finally, providers need to be able to adapt their examination skills for virtual care environments.

According to Edirippulige and Armfield [4,27], because using telehealth implies a change in practice, it should be supported by an appropriate level of evidence-based education for health care providers. An appropriate way to do this should start with educating and training future health care providers by incorporating telehealth education as a standard component in the prelicensure curriculum. At a CPD level, online education may be particularly attractive for busy practitioners who choose to participate in short CPD courses to develop knowledge and skills. However, it also seems that the practice of virtual care requires certain hands-on skills. Practical sessions can be helpful in developing such skills, as well as the observation of real-life or simulated virtual care appointments to gain exposure to the modality [22]. Our approach involves the development and provision of an accredited CPD e-learning module, designed to enhance the confidence and competencies of primary health care providers in virtual care adoption and use in their practices. An ongoing evaluation will be conducted with the findings used to improve e-learning approaches to teaching this important area for health care providers and health care delivery systems around the world.

The current evidence surrounding the most effective e-learning modalities is limited by the fact that the reported program designs differ with variation in the types of modalities used to deliver virtual care CPD. There are also limited studies on the effectiveness of asynchronous approaches like those described in this paper. This variation makes it difficult to draw conclusions around the most effective approach, although future comparative type studies could contribute to our understanding of the most effective approaches or combinations of modalities. Another notable observation of the existing literature is the general lack of evaluation at a “knowledge level.” Most evaluation studies have not reported assessment of knowledge as a key evaluative outcome from virtual care CPD, whether online or in person. Calleja et al [47] suggest this lack of standardized knowledge evaluation is common among virtual care training programs. The field would benefit from more consistent application of systematic evaluation frameworks, such as Kirkpatrick’s [48] or Moore et al’s [49] models of evaluation.

The need for virtual care is greater than ever, and health care providers must receive appropriate and meaningful education and training to understand the best ways to conduct virtual care appointments. The current evidence suggests online CPD approaches have been a more common approach, particularly during and after the COVID-19 pandemic. Online CPD on virtual care appears to have been well received by participants; however, there is a lack of evidence surrounding the effectiveness of interactive asynchronous online learning designs like that described in this paper. Asynchronous designs afford greater convenience and flexibility for providers in accessing CPD at times that are best for them. An adaptation of Kirkpatrick’s [46] levels of evaluation model is being applied to understand the effectiveness of our asynchronous design, and this will offer further evidence around this online learning modality for CPD on virtual care.

## Abbreviations

ADDIE: Analysis, Design, Development, Implementation, and Evaluation
AFMC: Association of Faculties of Medicine of Canada
CACME: Committee on Accreditation of Continuing Medical Education
CFPC: College of Family Physicians of Canada
CIHI: Canadian Institute for Health Information
CMA: Canadian Medical Association
CMPA: Canadian Medical Protective Association
CPD: continuing professional development
CRe-DEPTH: Criteria for Reporting on Development and Evaluation of Professional Training interventions in Healthcare
OPED: Office of Professional & Educational Development
PHP: Hypertext Preprocessor
RCPSC: Royal College of Physicians and Surgeons of Canada
SPC: scientific planning committee
